# Targeting novel structural and functional features of coronavirus protease nsp5 (3CL^pro^, M^pro^) in the age of COVID-19

**DOI:** 10.1099/jgv.0.001558

**Published:** 2021-01-28

**Authors:** Molly K. Roe, Nathan A. Junod, Audrey R. Young, Dia C. Beachboard, Christopher C. Stobart

**Affiliations:** ^1^​ Department of Biological Sciences, Butler University, Indianapolis, IN, USA; ^2^​ Department of Biology, DeSales University, Center Valley, PA, USA

**Keywords:** Coronavirus, nsp5, 3CLpro, inhibitors, protease

## Abstract

Coronavirus protease nsp5 (M_^pro^_, 3CL_^pro^_) remains a primary target for coronavirus therapeutics due to its indispensable and conserved role in the proteolytic processing of the viral replicase polyproteins. In this review, we discuss the diversity of known coronaviruses, the role of nsp5 in coronavirus biology, and the structure and function of this protease across the diversity of known coronaviruses, and evaluate past and present efforts to develop inhibitors to the nsp5 protease with a particular emphasis on new and mostly unexplored potential targets of inhibition. With the recent emergence of pandemic SARS-CoV-2, this review provides novel and potentially innovative strategies and directions to develop effective therapeutics against the coronavirus protease nsp5.

## Human coronaviruses, disease and the potential for future emerging coronaviruses

Coronaviruses are enveloped, positive-strand RNA viruses responsible for a wide range of diseases in a diverse range of animal hosts. To date, seven human coronaviruses (HCoVs) have been identified to cause respiratory diseases of varying severities: HCoV-OC43, HCoV-229E, HCoV-NL63, HCoV-HKU1, SARS-CoV, MERS-CoV and SARS-CoV-2. Among these seven HCoVs, four (HCoV-OC43, HCoV-229E, HCoV-NL63 and HCoV-HKU1) are common co-circulating, seasonal coronaviruses that cause mild respiratory tract infections generally associated with cases of the common cold [1–4]. While these viruses are capable of more severe illness in more sensitive and susceptible populations, such as those that are immunocompromised, young and elderly, limited attention or support was given to the development of therapeutics or vaccines for coronaviruses until the emergence of the first of three novel pathogens of more significant disease, SARS-CoV, in 2002 [2, 5].

SARS-CoV emerged in November 2002 in Guangdong Province, China, and ultimately spread to 29 countries, infecting over 8000 individuals, in a 2-year span [6, 7]. Severe acute respiratory syndrome (SARS) is characterized by flu-like symptoms including a high fever, myalgia, and in advanced forms of the disease commonly dyspnoea and pneumonia [8]. In contrast to the low mortality rates associated with the common cold-associated coronaviruses, SARS-CoV was the first of three HCoVs that illustrated the emerging potential for significant disease with a case fatality rate of approximately 10 % [9]. Approximately a decade after the SARS-CoV outbreak, a second emerging severe coronavirus pathogen, MERS-CoV, emerged in 2012 in Saudi Arabia [10]. Like SARS-CoV, this pathogen was capable of significant lower respiratory disease with flu-like symptoms leading to dyspnoea, pneumonia and acute respiratory distress syndrome (ARDS) [11, 12]. However, MERS-CoV was associated with a far greater potential of severe disease with a case fatality rate of approximately 35–40 % [12]. While both SARS-CoV and MERS-CoV have highlighted the potential for significant disease and mortality, public health efforts, barriers to human-to-human transmission and limited asymptomatic spread all hindered their pandemic potentials [13–15].

In December 2019, cases of viral pneumonia of an unknown cause first appeared in Wuhan, Hubei Province, China [16, 17]. Quickly recognized by the World Health Organization (WHO) as a ‘public-health emergency of international concern’ in January 2020 and later classified as a true pandemic, SARS-CoV-2 has resulted in over 67 million confirmed cases and 1.5 million deaths worldwide (as of December 2020) [18]. Studies of transmissibility of SARS-CoV-2 have indicated a greater R_0_ than SARS-CoV and MERS-CoV, suggesting a greater rate of transmissibility and infectivity for SARS-CoV-2 compared to either virus [19–22]. SARS-CoV-2 spreads mostly through respiratory droplets and direct contact with asymptomatic or symptomatic infected persons [20–23]. Although all three emerging infectious diseases share fever, shortness of breath and severe pneumonia as clinical manifestations, SARS-CoV-2 disease (named COVID-19) can specifically cause systemic inflammation which can develop further into acute cardiac injuries, sepsis, abnormal organ functions and heart failure [19, 23, 24]. Other distinctive clinical features of SARS-CoV-2 include sore throat, hypoxaemia, dyspnoea, sneezing and diarrhoea [22, 23]. Unlike SARS-CoV and MERS-CoV, SARS-CoV-2 has the potential to continue co-circulating throughout the world with the four common cold-associated HCoVs because of its lower case fatality rate and greater transmissibility [25].

Research suggests that all three emergent betacoronaviruses are zoonotic and originated within different bat species [26–28]. Due to infrequent bat–human interactions, the intermediate hosts responsible for SARS-CoV, MERS-CoV and SARS-CoV-2 acquiring the appropriate mutations to infect humans were probably palm civets, dromedary camels and potentially Malayan pangolins, respectively [1, 15, 27]. Even though SARS-CoV-2 shares a considerable amount of nucleotide sequence with MERS-CoV (51.8 %) and SARS-CoV (79.0 %), it is most similar (with greater than 95 % identity) to coronaviruses found in bats [20, 25, 27, 28]. There remain many more bat coronaviruses which have been identified and thus lack a human analogue, highlighting the continued potential for future emergent HCoVs.

Until recently, there remained no commercially available vaccines for coronaviruses and limited therapeutic options. Despite extensive investigation and hundreds of studies evaluating critical viral targets including the polymerase (nsp12; RdRp) and main protease (nsp5), there remains a critical need for the development of effective therapeutics to treat current and future coronavirus infections [29, 30].

### Coronavirus replication and the role of nsp5 protease

**Fig. 1.**
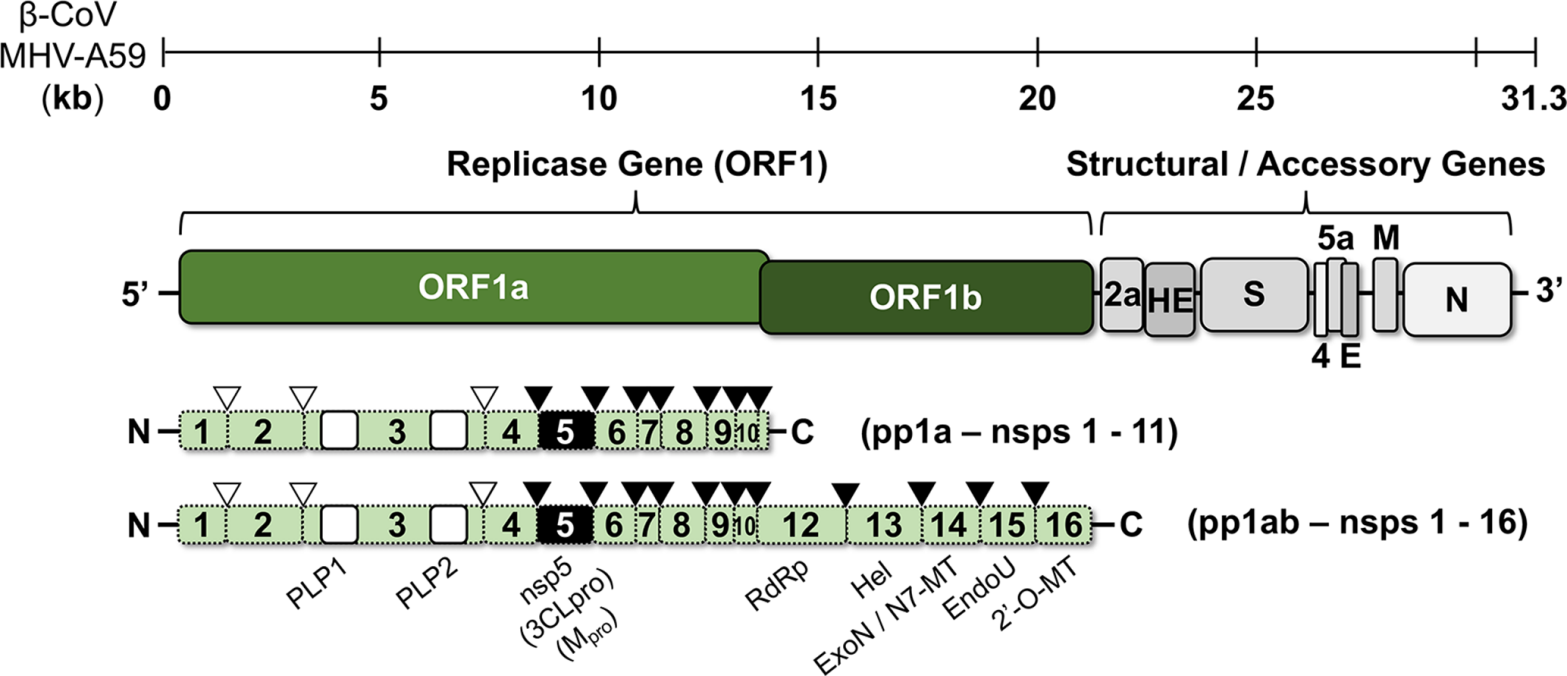
Coronavirus genome organization, replicase gene expression and polyprotein processing. The 31.3 kb genome of beta-coronavirus MHV-A59 is depicted. The viral ORFs associated with replication (replicase gene; ORF1a/ORF1b) and structural and accessory genes are shown. The two variant polyproteins (pp1a and pp1ab) translated from the replicase gene are shown with the non-structural protein domains of the polyprotein labelled and the proteolytic cleavage sites marked with arrows. Three proteases mediate the proteolytic processing of the replicase polyproteins [PLP1, PLP2 and nsp5 (3CL^pro^/M^pro^)], and the colour of the arrows (white for PLPs and black for nsp5) for each cleavage site correspond to the protease responsible for mediating its cleavage. PLP, papain-like protease; RdRp, RNA-dependent RNA polymerase; Hel, helicase; ExoN, exonuclease; N7-MT, N7-methyltransferase; EndoU, endoribonuclease; 2′-O-MT, 2′-*O*-methyltransferase.

Coronaviruses are enveloped viruses with 27–32 kb positive ssRNA (+ssRNA) genomes which are classified in four different genera (*Alpha*-, *Beta*-, *Gamma*- and *Deltacoronavirus*) within the order *Nidovirales* and family *Coronaviridae* [31]. During virus infection, coronaviruses employ trimeric spike (S) proteins to facilitate entry into host cells [32, 33]. The interaction of this protein with its receptor dictates species and tissue tropism. Among human coronaviruses, several different cellular fusion receptors have been identified, including aminopeptidase N (HCoV-229E), angiotensin-converting enzyme 2 (HCoV-NL63, SARS-CoV and SARS-CoV-2), and dipeptidyl peptidase 4 (DPP4) [34–38]. Upon receptor binding, the viral and cellular membranes are fused together triggered by spike (S) activation through proteolytic cleavage by a cellular protease such as TMPRR2 or cathepsin [39]. Immediately upon entry, the virus translates its replicase gene (ORF1) which consists of two large, overlapping ORFs, ORF1a and ORF1ab (Fig. 1) [31]. Located at the end of ORF1a, a ribosome frame-shifting sequence consisting of an RNA pseudoknot causes the co-translation of two large polyprotein precursors of differing lengths, pp1a and pp1ab [31, 40, 41]. Polyprotein pp1a contains non-structural proteins (nsps) 1–11, and polyprotein pp1ab comprises the complete translated coding region of nsps 1–16 [42, 43]. Essential for virus replication is the proteolytic processing of these polyproteins by virus-encoded proteases to yield the mature and functionally active replication machinery of the virus [42].

Once proteolytically processed, the translation products of pp1a collectively modulate host cell factors and help prepare the cell for viral RNA synthesis through the formation of replication complexes, while the C-terminal translation products of pp1ab largely catalyse and/or regulate the processes of RNA replication and transcription driven by the viral RdRp (nsp12) [31, 44]. Replication complexes assemble on virus-induced membrane structures such as double-membrane vesicles and convoluted membranes driven by transmembrane nsps 3, 4 and 6 [45–48]. The active replication complex promotes the continuous and discontinuous synthesis of negative-sense RNA templates, which are subsequently used to drive formation of genomic copies and a nested set of subgenomic RNAs from the downstream ORFs encoding structural and accessory proteins, respectively [49]. Following replication of genomic and subgenomic RNA on double-membraned vesicles, structural proteins like the spike (S), envelope (E), matrix (M) and nucleocapsid (N) proteins are translated by existing positive-strand subgenomic RNAs. S, E and M become glycosylated within the Golgi before localizing to the endoplasmic reticulum-Golgi intermediate compartment (ERGIC) to be assembled into virions [50–53]. The N protein will localize with the replicase proteins at replication complexes within the cytoplasm while RNA is synthesized, where it is thought to encapsidate the newly made RNA [53, 54]. After RNA synthesis, genomic RNA and N protein move to the ERGIC and assimilate into budding virions. Additional S protein is expressed on the cell surface where it triggers cell-cell fusion between infected cells and nearby, uninfected cells [55]. Consequently, massive, multinucleated cell complexes called syncytia often form, facilitating spread of the virus while avoiding neutralization via virus-specific antibodies [31, 55].

As earlier mentioned, proteolytic processing acts as a key regulatory mechanism in the expression of the coronavirus replicase proteins, as blocking this process has been demonstrated to block viral replication entirely [42, 56–58]. Typically, coronaviruses code for two or three proteases to process the replicase polyprotein: one or two papain-like proteases (PLPs) encoded within nsp3, and one main protease, nsp5 (3CLpro or M^pro^) [31]. PLPs are responsible for cleavage events between nsp1 and the N terminus of nsp4, whereas all remaining pp1a/pp1ab cleavage events are mediated by nsp5 [42, 59–61]. In addition, both proteases have been implicated in targeting host cell targets including modulating deubiquitination, deISGylation and virus evasion of the innate immune response [60, 62].

### Nsp5 protease structure and function

**Fig. 2.**
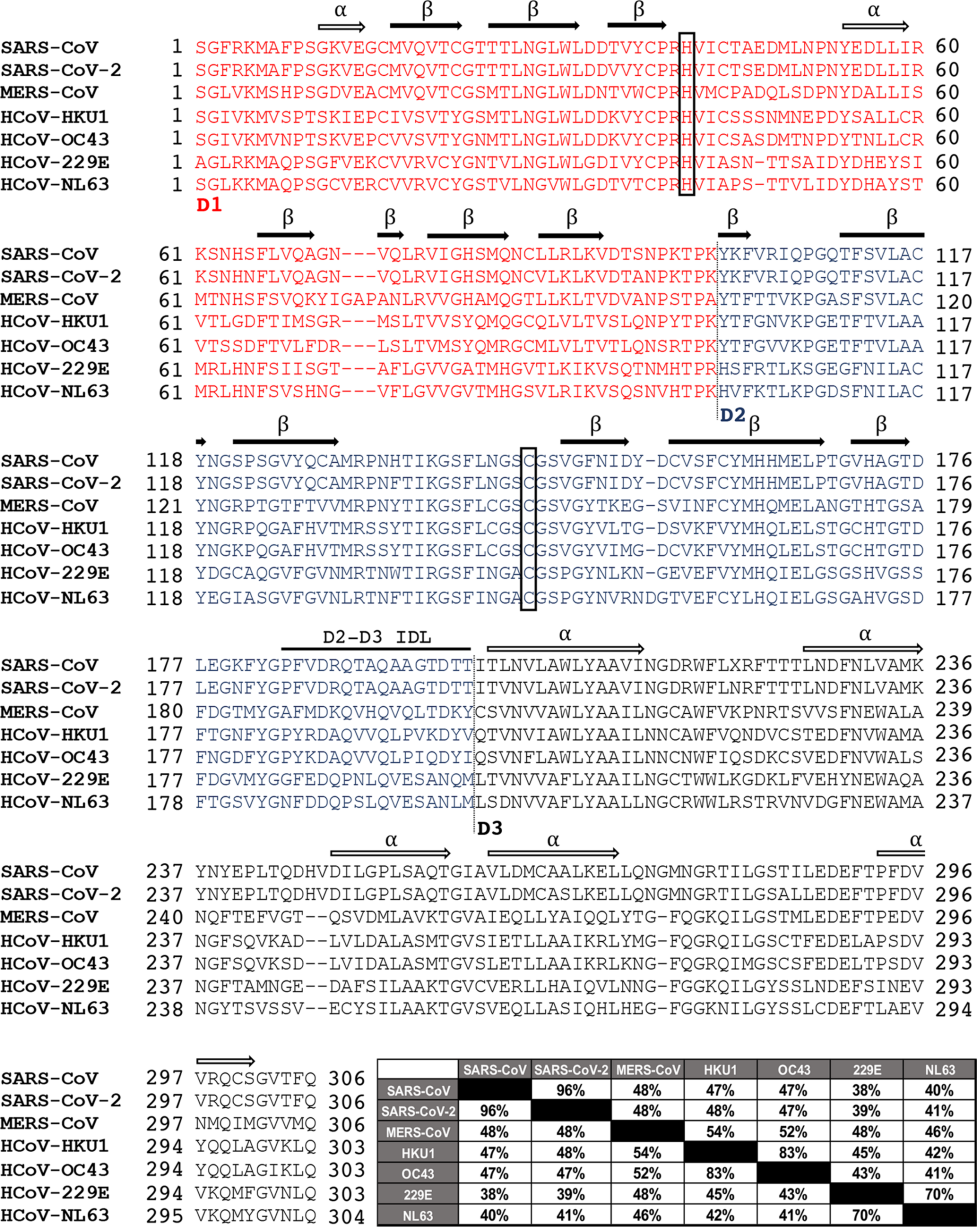
Sequence alignment and conservation of the nsp5 (3CL^pro^) protease sequences of the seven known human coronaviruses. The seven human coronavirus protease sequences were aligned and the three domains of the proteases are colour coded and labelled. Alpha helices (open arrows) and beta sheets (filled arrows) are shown and the catalytic dyad residues (His/Cys) are boxed. A conservation matrix depicting percentage identity between amino acid sequences is shown at the end of the sequence alignment.

**Fig. 3.**
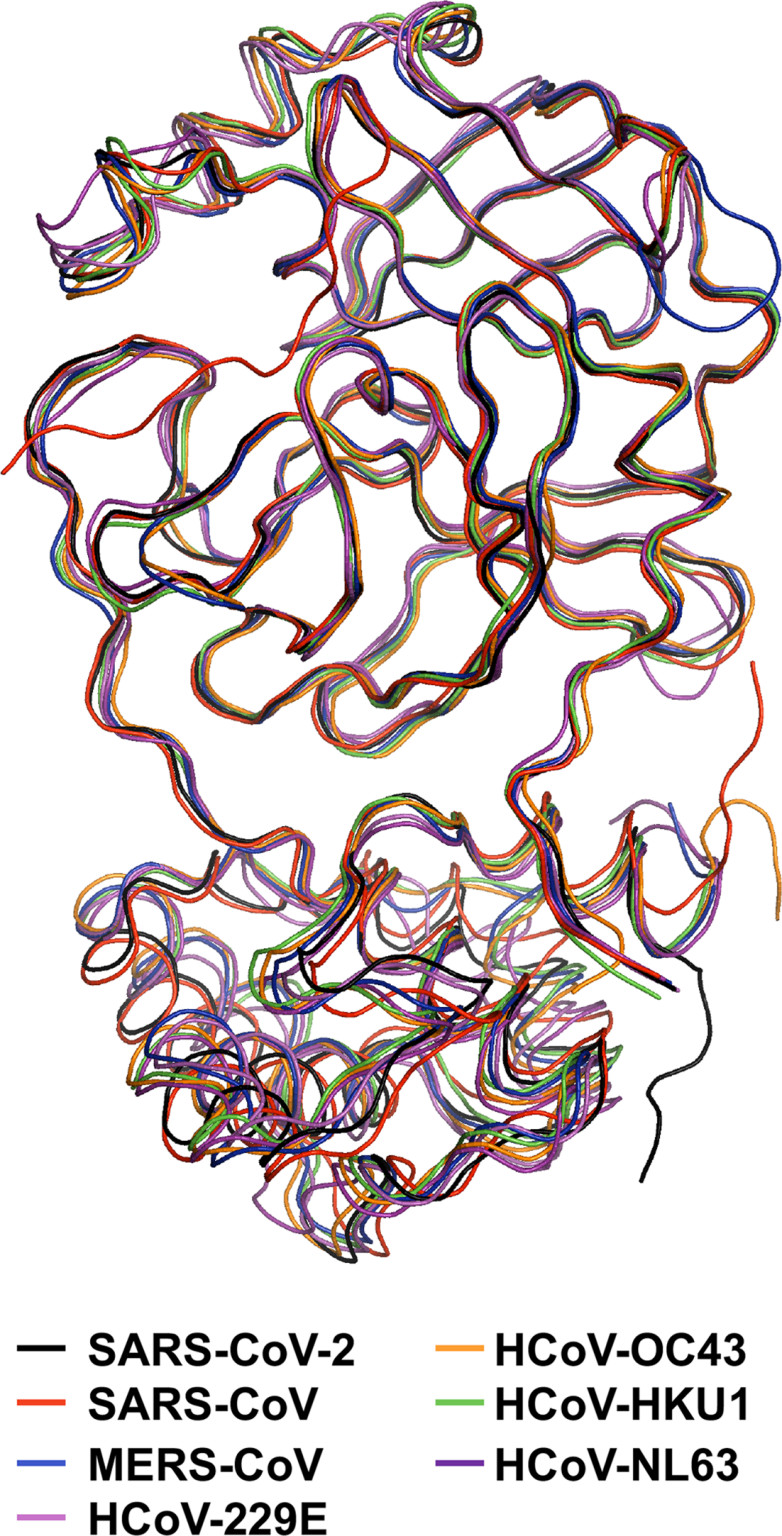
Structural alignment of HCoV nsp5 (3CL^pro^) protease crystal structures. An overlay of the monomeric crystal structures of HCoV nsp5 proteases of SARS-CoV-2 (PDB 6M2N), SARS-CoV (PDB 2Q6G), MERS-CoV (PDB 4YLU), HCoV-229E (PDB-2ZU2), HCoV-HKU1 (PDB-3D23), HCoV-NL63 (PDB-3TLO) and the modelled structure of HCoV-OC43 (PM0079872) [66, 75, 104, 139–143].

The coronavirus protease nsp5 (3CL^pro^ or M^pro^) is an approximately 30 kDa, three-domain cysteine protease conserved in structure and function in all known coronaviruses and serves as the main protease for proteolytic processing of the replicase polyproteins (pp1a and pp1ab) [31, 42, 63, 64]. The name ‘main protease’, or M^pro^, refers to the critical role of this protease in coronavirus gene expression and replicase processing, and its other name ‘3C-like protease’ (3CL^pro^) refers to the similarities between this protease and 3C proteases seen in picornaviruses, namely their similar substrate specificities and core structural homology [65]. Among coronaviruses, nsp5 proteases within the same genus generally exhibit sequence identity of greater than 80 % whereas protease in different genera are far more divergent with sequence identity much closer to 50 % despite high tertiary and quaternary structural conservation especially in domains 1 and 2 (Fig. 2). Unsurprisingly, the greatest degree of sequence conservation exists in and around the enzyme active site (Fig. 3). Sequence analysis of the SARS-CoV and SARS-CoV-2 proteases reveals only 12 residue differences (approximately 96 % identity) spread throughout the structure of the protease, with the majority of these residues distant from the active site (including along the distal surface of domain 1 and within domain 3), which strongly supports the prospect of developing active-site inhibitors that target both proteases.

**Fig. 4.**
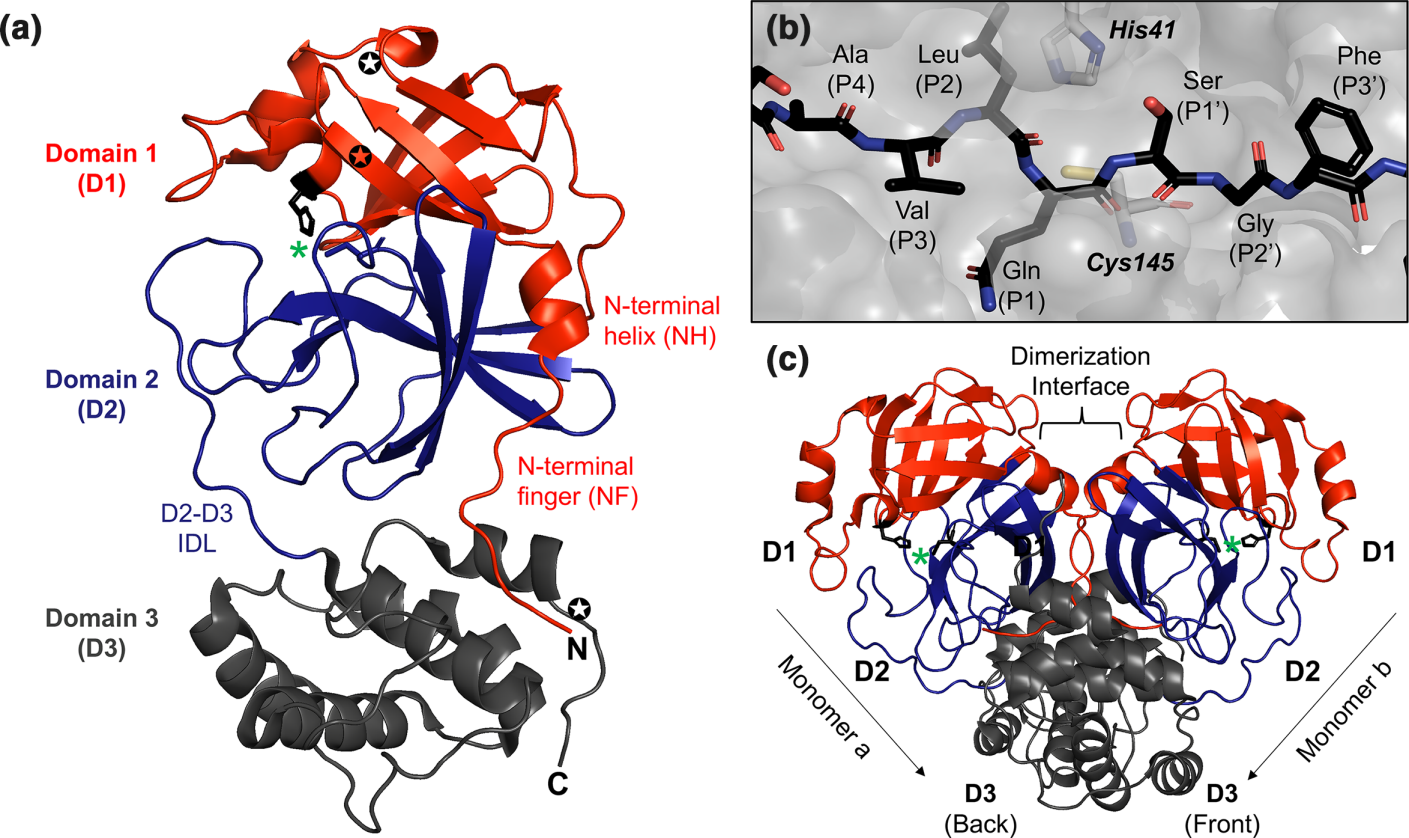
Nsp5 (3CLpro) structural features of SARS-CoV-2 protease. (a) Monomeric structure of the SARS-CoV-2 nsp5 protease (PDB 6M2N) with the three domains shown. Key structural regions including the N-terminal finger (NF), N-terminal helix (NH) and domain 2–domain 3 interdomain loop (IDL) are labelled. Corresponding locations of MHV nsp5 resistance mutations identified in Deng *et al*. are labelled with stars [144]. (b) A view of the active site with a consensus cleavage site peptide bound. The catalytic dyad residues are shown. (c) SARS-CoV-2 nsp5 dimer structure with the orientation of the two dimers shown by arrows and the dimerization interface labelled. Green asterisks denote the catalytic site with black sticks shown for the catalytic dyad residues (His41 and Cys145) in both the monomeric (a) and the dimeric structures (c).

The N-terminal domains (1 and 2) of nsp5 are structurally highly conserved and form a chymotrypsin-like fold consisting of beta-barrels which surround the substrate binding site between the two domains (Fig. 4) [63, 65, 66]. The catalytic activity of the protease is mediated by a His-Cys catalytic dyad with the Cys residue serving as a nucleophile in the enzyme-catalysed proteolytic reaction. The nsp5 protease almost exclusively orchestrates cleavage after a P1-Gln (with few exceptions) [67–70]. The P2 substrate residue is also generally well conserved with typically a Leu, although other residues can occupy this site including Met, Phe, Val or Ile [67, 69]. The P1’ substrate residue typically shows much more diversity with Ser, Gly, Ala and Val residues all being found in known nsp5 cleavage sites. Far more diversity in amino acid residue usage is observed at the P2–P5 and P2’–P3’ sites. Collectively, the consensus cleavage site for nsp5 across known coronaviruses is P3-PLQ-(S/G/A/V)-P1’ [67]. The high specificity and consistency in cleavage site recognition among known coronaviruses has made the enzymatic active site of nsp5 the primary target for current inhibitor design efforts (Fig. 3).

While nsp5 domains 1 and 2 have been well characterized, much less is known of the role of the alpha-helical third domain of the protease. Most of the function of the helical third domain of nsp5 has been shown to direct nsp5 dimerization and help stabilize the chymotrypsin-like fold [71, 72]. Nsp5 dimerization is essential for nsp5 function and the monomeric form of the protease is largely inactive [65, 73–75]. In the nsp5 dimer, the monomers of the protease are orientated with their active sites facing away from one another with their N-terminal extensions (N-terminal fingers) and third domains directly interacting along a dimerization interface (Fig. 4). Studies evaluating residues important for dimerization have demonstrated that Glu166 in SARS-CoV forms critical interactions with the N-terminal finger residues of the heterologous monomer [68, 76]. Deletion of the first few residues in the N-terminal finger of SARS-CoV results in significant losses in enzymatic activity and disruption of dimerization [68, 77]. Structural and biochemical studies have demonstrated that subtle differences exist in the coordinating residues for dimerization between SARS-CoV, MERS-CoV and SARS-CoV-2 [75, 78]. In addition, it has been shown that dimerization in MERS-CoV requires ligand association, whereas such a requirement is not present for SARS-CoV [75]. Prior to processing, nsp5 is found within a >800 kDa precursor which is processed into a 150 kDa, nsp4–10 precursor [79, 80]. nsp5 is initially responsible for coordinating its own autoproteolytic cleavage [68, 81]. It is hypothesized that two nsp5 proteases anchored to membranes by transmembrane protein nsp4 and nsp6 form a dimer and trigger cleavage *in trans* [68, 75, 81, 82]. Upon its own maturation cleavage, nsp5 is believed to target the nsp9–10 site, prior to targeting the nsp8–9 and nsp7–8 sites, respectively, for processing [83]. Once these sites are processed, the other nsps that nsp5 is responsible for cleaving are individually separated from the nsp7–10 site. Before the final processing of nsps, one of the intermediate complexes, nsp7+8, conducts an important function: catalysing the cleavage of nsp12, an essential viral polymerase. Studies involving mutation of the nsp5 cleavage sites have shown that disruption of the nsp7–nsp8 and nsp8–nsp9 cleavage sites results in loss of virus viability whereas other sites such as the nsp9–nsp10 site can be tolerated with reduced replication [84]. The ordered processing by the nsp5 protease may represent a unique facet of viral replication that may be disrupted by inhibitors.

It has been suggested that nsp5 associates with numerous other components of the replication complex. Several studies have shown important intra- and intermolecular associations between the nsp5 protease and the rest of the replicase gene, with mutations both within the nsp5 domain and mutations in nsp3 and nsp10 negatively impacting nsp5 activity [80, 85, 86]. These data strongly suggest that important allosteric interactions exist between nsp5 and other members of the replicase gene. In addition, several temperature-sensitive mutations have been identified within the nsp5 proteases of mouse hepatitis virus (MHV) and HCoV-OC43 that selected for second-site compensatory mutations that were distant (>15 Å) from the initial mutation site [63, 85]. These data indicate that complex interactions which span all three domains of the protease are critical for protease structure and function. Additional studies are needed to understand their role as they may represent novel directions for proteolytic inhibition.

### Therapeutic design strategies for nsp5 inhibitors

As SARS-CoV-2 continues to spread and interfere with our daily lives, a need for an effective, safe way to treat the viral infections has become much more prevalent. While the efficacy and safety of an array of vaccine candidates is currently being evaluated, it seems unlikely that worldwide distribution of available vaccines at rates needed for herd immunity will not occur for quite some time [87, 88]. In addition to the challenges of testing for safety and efficacy, vaccines target specific pathogens which make them limited in treating future problematic diseases, especially diseases caused by rapidly mutating and evolving RNA coronaviruses. Because of these concerns, the practicality of utilizing therapeutic alternatives to treat current and future viral outbreaks appears more promising [87]. Considering the heightened interactions among humans and animals, the immense viral diversity characterizing coronaviruses, and frequent adaptations and mutations, targeting a conserved region among coronaviruses for therapeutic treatment might de-escalate the lingering threat of future human coronavirus outbreaks or pandemics [13].

Since the SARS-CoV outbreak of 2003–2004, there have been ongoing efforts to develop inhibitors that target nsp5. Several compounds have been designed and tested for nsp5 active-site inhibition, including esters and covalent or non-covalent peptidomimetics (summmarized in Table 1) [89, 90]. The covalent modifiers (esters and peptidomimetics) act as competitive inhibitors that mimic natural peptide substrates of nsp5. The non-covalent inhibitors occupy the substrate binding site to competitively inhibit nsp5 activity. Additionally, some groups have done computational screening of known drug libraries to identify potential inhibitors [91, 92].

**Table 1.** Select nsp5 inhibitors

Compound	Type of compound/source	Virus	IC_50_ (μM)	EC_50_ (μM)	References
CE-5	Chloropyridine-esters	MHV	nd	8.5	[94]
		SARS-CoV	nd	24±0.9	[95]
		MERS-CoV	nd	13.5	[96]
18a	Peptidomimetic – AG7088 derivative	SARS-CoV	10	18.86	[101]
18b	Peptidomimetic – AG7088 derivative	SARS-CoV	5	9.45	
18c	Peptidomimetic – AG7088 derivative	SARS-CoV	1	0.18	
18d	Peptidomimetic – AG7088 derivative	SARS-CoV	10	0.11	
18e	Peptidomimetic – AG7088 derivative	SARS-CoV	7	0.16	
1	Peptidomimetic – AG7088 derivative	SARS-CoV	870/45	nd	[98]
2	Peptidomimetic – AG7088 derivative	SARS-CoV	800/70	nd	
3	Peptidomimetic – AG7088 derivative	SARS-CoV	8075	nd	
4	Peptidomimetic – AG7088 derivative	SARS-CoV	10/100	nd	
5	Peptidomimetic – AG7088 derivative	SARS-CoV	15/100	nd	
16	Peptidomimetic – AG7088 derivative	SARS-CoV	300/nd	nd	
17	Peptidomimetic – AG7088 derivative	SARS-CoV	200/nd	nd	
4a	Peptidomimetic	SARS-CoV MERS-CoV	>25 >25	nd nd	[145]
5a	Peptidomimetic	SARS-CoV MERS-CoV	>25 >25	nd nd	
6a	Peptidomimetic	SARS-CoV MERS-CoV	>25 >25	nd >100	
6b	Peptidomimetic	SARS-CoV MERS-CoV 229E OC43 FIPV	0.7±0.2 2.4±0.3 nd nd nd	nd 1.4±0.0 4.3±0.1 13.5±0.8 2.5±1.1	
6c	Peptidomimetic	SARS-CoV MERS-CoV 229E OC43 FIPV	0.5±0.1 4.7±0.6 nd nd nd	nd 1.2±0.6 4.2±0.3 16.8±0.3 1.9±0.2	
6d	Peptidomimetic	SARS-CoV MERS-CoV 229E OC43 FIPV	nd 1.7±0.3 nd nd nd	nd 0.6±0.0 2.0±0.2 17.7±1.6 1.1±0.3	
9a	Structure-based design	MERS SARS FIPV	0.6 2.1 0.8	nd	[103]
10a	Structure-based design	MERS SARS FIPV	0.4 5.1 2.4	0.5 0.6 1.5	
9b	Structure-based design	MERS SARS FIPV	0.7 28.8 3.5	nd	
10b	Structure-based design	MERS SARS FIPV	0.6 42.1 2.3	nd	
9c	Structure-based design	MERS SARS FIPV	0.8 5.2 1.6	nd	
10c	Structure-based design	MERS SARS FIPV	0.7 6.3 2.1	0.8 1.0 0.1	
9d	Structure-based design	MERS SARS FIPV	0.7 3.9 0.8	nd	
10d	Structure-based design	MERS SARS FIPV	0.9 4.3 1.1	nd	
9e	Structure-based design	MERS SARS FIPV	6.1 5.5 5.5	nd	
10e	Structure-based design	MERS SARS FIPV	7.5 4.1 6.7	nd	
9f	Structure-based design	MERS SARS FIPV	0.6 3.2 1.3	nd	
10f	Structure-based design	MERS SARS FIPV	0.5 8.8 1.1	nd	
11a	Structure-based design	SARS-CoV HCoV-NL63	1.95±0.24 >50	2.0±0.2	[106]
11n	Structure-based design	SARS-CoV HCoV-NL63	0.33±0.04 1.08±0.09	7.2±0.2	
11r	Structure-based design	SARS-CoV HCoV-NL63	0.71±0.36 12.27±3.56	1.4±0.1	
11s	Structure-based design	SARS-CoV HCoV-NL63	0.24±0.08 1.37±0.35	1.9±0.1	
11t	Structure-based design	SARS-CoV HCoV-NL63	1.44±0.4- 3.43±2.45	6.7±0.2	
11u	Structure-based design	SARS-CoV HCoV-NL63	1.27±0.34 5.41±2.31	3.6±0.1	
48	Peptidomimetic-decahydroisoquinolin derivatives	SARS-CoV	68	nd	[109]
41	Peptidomimetic-decahydroisoquinolin derivatives	SARS-CoV	63	nd	
49	Peptidomimetic-decahydroisoquinolin derivatives	SARS-CoV	49	nd	
6n	Peptidomimetic -serine derivatives	SARS-CoV	85	nd	[1]
6o	Peptidomimetic -serine derivatives	SARS-CoV	65	nd	
4 (SK80)	Phenylisoserine derivatives	SARS-CoV	43	nd	[111]
10	Peptidomimetic – phenylisoserine derivatives	SARS-CoV	85	nd	
17	Peptidomimetic – phenylisoserine derivatives	SARS-CoV	75	nd	
18	peptidomimetic - phenylisoserine derivatives	SARS-CoV	65	nd	
12	Peptidomimetic – phenylisoserine derivatives	SARS-CoV	65	nd	
Ac-Val-Leu-NHCH(CH2CH2CON(CH3)2)-CHO	Peptidomimetic	SARS-CoV	~6	nd	[108]
Ac-Ser-Ala-Val-Leu-NHCH(CH2CH2CON(CH3)2)-CHO	Peptidomimetic	SARS-CoV	37	nd	
Ac-Thr-Ser-Ala-Val-Leu-NHCH(CH2CH2CON(CH3)2)-CHO	Peptidomimetic	SARS-CoV	26	nd	
Cinanserin	*In silico* design	SARS-CoV HCoV-229E	4.92 4.68	nd	[112]
Cinanserin hydrochloride	*In silico* design	SARS-CoV HCoV-229E	5.05 5.68	nd	
53	Non-covalent	SARS-CoV	10	nd	[113]
54	Non-covalent	SARS-CoV	5.5	nd	
56	Non-covalent	SARS-CoV	45	nd	
10c	Non-covalent	SARS-CoV	11	nd	[114]
13a	Non-covalent	SARS-CoV	7.72	nd	
13b	Non-covalent	SARS-CoV	25.3	nd	
13c	Non-covalent	SARS-CoV	6.9	nd	
13d	Non-covalent	SARS-CoV	4.1	nd	
13e	Non-covalent	SARS-CoV	22.5	nd	
13f	Non-covalent	SARS-CoV	9.1	nd	
13g	Non-covalent	SARS-CoV	3.8	nd	
13k	Non-covalent	SARS-CoV	26	nd	
16a	Non-covalent	SARS-CoV	2.9	nd	
16b	Non-covalent	SARS-CoV	3.6	nd	
16c	Non-covalent	SARS-CoV	13.3	nd	
16d	Non-covalent	SARS-CoV	3.4	nd	
16e	Non-covalent	SARS-CoV	4.1	nd	
16f	Non-covalent	SARS-CoV	8.1	nd	
16g	Non-covalent	SARS-CoV	22.1	nd	
16i	Non-covalent	SARS-CoV	10.3	nd	
16j	Non-covalent	SARS-CoV	2.1	nd	
16k	Non-covalent	SARS-CoV	1.5	nd	
17a	Non-covalent	SARS-CoV	0.051	nd	
17b	Non-covalent	SARS-CoV	0.97	nd	
17c	Non-covalent	SARS-CoV	0.70	nd	
17d	Non-covalent	SARS-CoV	2.0	nd	
17e	Non-covalent	SARS-CoV	15.5	nd	

nd, not determined.

The first ester-based compounds to be developed were benzotriazole ester-based and irreversibly acylated the C145 within the active site [93]. Next, chloropyridine-esters were tested and found to have potent inhibitory activity against nsp5. CE-5 is one of the chloropeptidyl esters that has been extensively studied and shown to have broad activity across several CoVs. CE-5 has an EC_50_ of 8.5 µM for MHV [94], 24±0.9 µM for SARS-CoV [95] and 13.5 µM for MERS-CoV [96]. Additionally, this inhibitor was tested during BtCoV-HKU5 and MERS-CoV infection, where it resulted in a 10–100-fold decrease in viral titres depending on the timepoint [97].

Extensive work has been done to identify peptidomimetic inhibitors of nsp5 [89]. The majority of these compounds contain Michael acceptors, chloromethyl ketone or epoxide functional groups. These functional groups form covalent, irreversible interactions with nsp5 C145. However, some of these compounds also form non-covalent interactions with the nsp5 active site. Early peptidomimetic inhibitors were designed based on AG7088, which targets the human rhinoviral 3C protease [98, 99]. While the AG7088 compound was not effective in SARS-CoV-infected cells [100], it was used to subsequently design molecules that have antiviral activity against the SARS-CoV nsp5 protease. Two groups studied the AG7088 derivative and showed potent activity on the SARS-CoV nsp5 [98, 99, 101]. Shie *et al*. identified five compounds with EC_50_ values of <20 µM, three of which were <0.2 µM. Kumar and colleagues extended the testing of 3C^pro^ inhibitors against both SARS-CoV and MERS-CoV and identified compounds with low micromolar activity and EC_50_ of 0.6–1.4 µM [102].

In addition to testing known inhibitors of 3C proteases, a structure-based design has also been used to identify nsp5 inhibitors. Using the crystal structure of MERS-CoV in complex with the antiviral compound GC376, Galasiti Kankanamalage *et al.* designed several compounds and measured the IC_50_ for nsp5 of MERS-CoV, SARS-CoV and feline infectious peritonitis virus (FIPV) [103]. Interestingly, some of the compounds had varying degrees of activity against the different proteases. For the compounds tested, the MERS-CoV IC_50_ ranged from 0.5 to 7.5 µM, FIPV ranged from 0.8 to 6.7 µM, but SARS-CoV was more divergent with ranges from 2.1 to 42.1 µM. They then tested the EC_50_ of two compounds with MERS-CoV, MHV and FIPV and showed a range of 0.5–1.5 µM across the three viruses. However, SARS-CoV was not tested for EC_50_ and it had the highest, most divergent IC_50_ values among the viruses tested. These data probably indicate differences in binding within the active site of each of these proteases and highlights the need to test inhibitors against multiple CoV proteases. This finding is consistent with both variations in the active sites between the CoV proteases (Fig. 2) as well as known differences in cleavage site sequences in pp1ab [67]. Consistent with numerous other studies evaluating inhibitors bound to crystallized nsp5 proteases, coordination between the active site inhibitor and subtle variations in the residues lining the pocket may be impacting the strength of binding and fit of the inhibitor [75, 104, 105]. In another study using a structure-based design, Zhang *et al.* were able to develop several α-ketoamides that have a glutamine lactam at the P1 site that mimics the glutamine in the P1 position of the nsp5 consensus cleavage site [106]. This strategy resulted in the design of six compound derivatives with EC_50_ values of <10 µM against SARS-CoV replicons and cross-reactivity with the enterovirus EV-A71 and CVB3 3C proteases. This cross-reactivity would allow for development of more broad-spectrum antivirals that can be tested using structure–activity relationships (SARs) to make the compound more specific and potent to certain viral proteases.

There have been challenges to screening compounds *in vitro* with tagged nsp5. While purifying N-terminally tagged nsp5 for testing peptidomimetic inhibitors, Akaji *et al.* identified an internal cleavage site in the SARS-CoV nsp5 at R188-Q189 that resulted in loss of enzyme activity that could have obscured the results of inhibition activity. They introduced an R188I mutation that prevents internal cleavage during *in vitro* screening and then used a structure-based design of inhibitors. Through several follow-up studies, they have identified inhibitors based on several scaffolds including aldehyde, decahydroisoquinolin, and serine or phenylisoserine derivatives. Using SAR analysis, they were able to identify compounds with IC_50_ of <100 µM [107–111]. In future studies, this R188I substitution will need to be used to screen compounds if tagging the N terminus of nsp5.

There has been some computational analysis of potential nsp5 inhibitors. Chen *et al.* screened the MDL-CMC database for inhibitors that bound the nsp5 active site *in silico* [112]. They then identified 10 compounds and were able to show that cinanserine had antiviral activity against nsp5 from both SARS-CoV and HCoV-229E, as well as the HCoV-229E replicon cells, and during infections with SARS-CoV and HCoV-229E. However, a subsequent study showed no inhibition of the SARS-CoV, HCoV-229E or MHV nsp5 proteases up to 100 µM of cinanserine [113]. Jacobs *et al.* used a high-throughput assay to test several non-covalent nsp5 inhibitors and identified several with an IC_50_ of >100 µM and showed that ML-188 [also called 16-(R)] had an EC_50_ between 12.9±0.7 and 13.4±1.0 µM. Subsequently, this group then screened additional compounds and performed SAR studies to improve the efficacy of the compounds toward the SARS-CoV nsp5 protease [114].

### Novel potential targets for future nsp5 inhibitor design

**Fig. 5.**
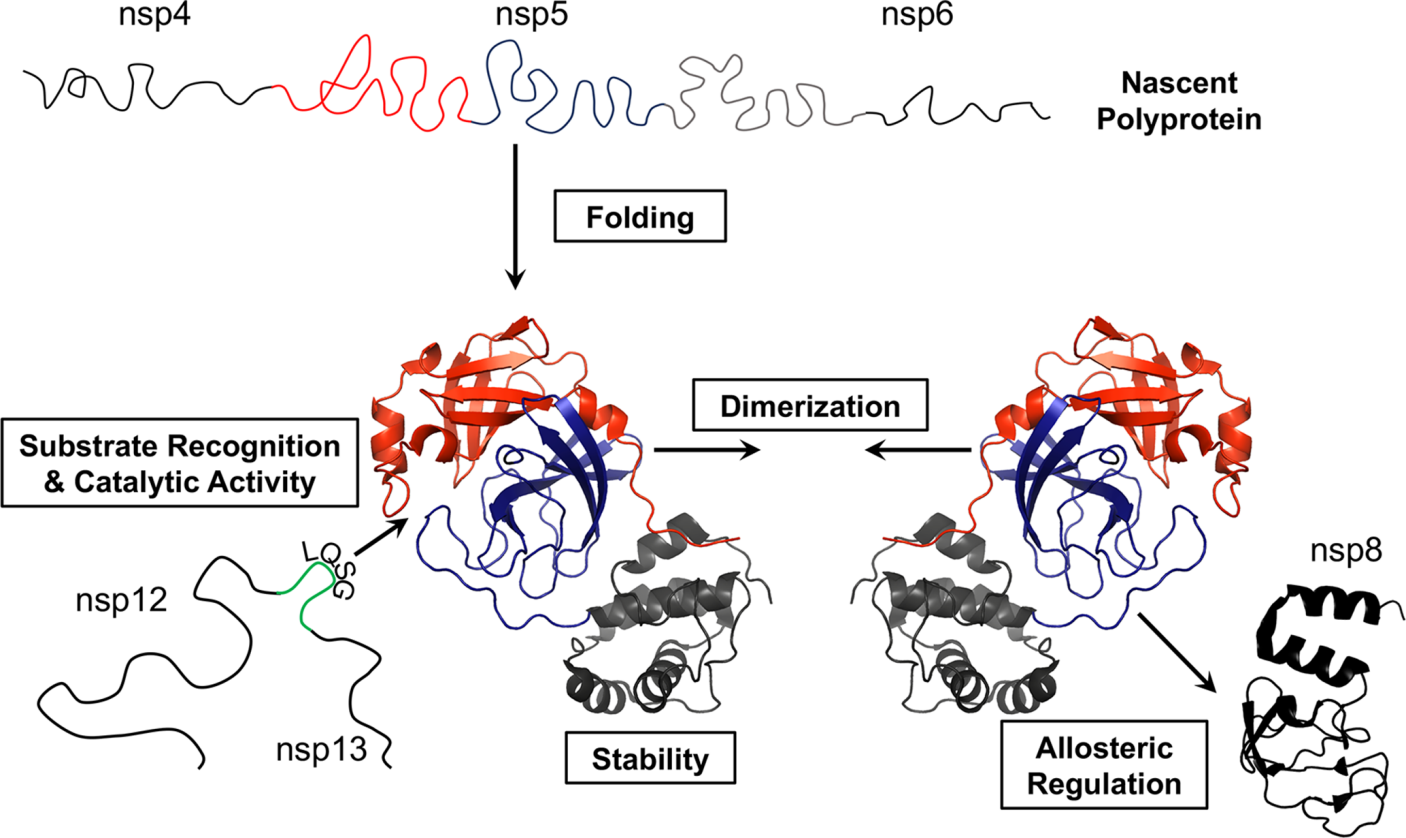
Potential targets for coronavirus nsp5 therapeutic design. An outline of the key stages of nsp5 activity is shown. Crystal structures of monomeric SARS-CoV-2 nsp5 (PDB 6M2N) and the C-terminal region of the SARS-CoV-2 nsp8 structure (PDB 7BV1) are also depicted [146].

There has been limited work on testing for resistance to these active-site inhibitors. However, one study showed that three substitutions arose that block inhibition of CE-5 [94]. Of the three substitutions, two were in domain 1 and the other was in domain 3 (Fig. 4). Interestingly, the substitutions in domain 1 were not in the active site but were located above the active site. This, combined with studies demonstrating that temperature-sensitive mutants were capable of gaining function through substitutions in distant portion of the proteins [85, 115], suggests that (1) we should expect that resistance mutations will arise to these active site inhibitors, and (2) those changes that confer resistance may be in one of many different locations throughout the protease due to the complex interactions within the protease that are required for enzymatic function and between the protease and host and viral proteins regulating its function allosterically. Therefore, targeting multiple regions of the protease may be a better strategy than using a single therapeutic that targets the active site. Therefore, there remain many additional potential approaches which may be used to inhibit nsp5 as highlighted in Fig. 5, which include protein folding and stability, dimerization, and allosteric interactions with host and viral proteins.

### Protein folding and stability

In order to be catalytically active, nsp5 has to be properly folded into the correct conformation after it is cleaved from the polyprotein. While it may not be possible to target specific folding of nsp5, there has been studies showing the use of protein folding inhibition in cancer treatment [116]. Since this would alter global protein folding, there may be challenges in delivery of such a compound. As groups are screening compounds *in silico*, it would be interesting to also look for allosteric inhibitors that bind to nsp5 and alter the conformation of the active site to preclude substrate binding or alter protein stability. The interdomain loop (IDL), which connects domains 2 and 3, is a structurally conserved region of the protease that is probably involved in substrate recognition [78, 117–119]. This region would be an interesting target for inhibition since altering the IDL could change the stability of the chymotrypsin-like fold or alter substrate recognition to decrease enzymatic activity. Other regions are known to be involved in protein stability and function. Through the study of nsp5 temperature-sensitive mutants and their second-site suppressor mutations in both MHV and OC43 [63, 85], it has been suggested that there are long-distance communication networks within the nsp5 structure. These nodes of the long-distance communication can be targeted by small molecules to alter the conformation and stability of nsp5.

### Dimerization

Targeting dimerization of a protease is a strategy that has been explored with human immunodeficiency virus (HIV) [120–122]. The mechanism of inhibition is to design peptides that match the dimerization region that will then bind to the region and prevent the binding of a second protease molecule. Dimerization of nsp5 is critical for nsp5 function [123]. Based on the strategy used for the HIV protease, an N-terminal octapeptide of nsp5 has been designed and shown to inhibit dimerization [124, 125]. This peptide is likely to block interaction of the N-finger regions to prevent dimerization. Additional studies could be performed to enhance the potency of the peptide inhibitors of dimerization.

### Allosteric interactions with host and viral proteins

Nsp5 interacts with other viral and host proteins to mediate virus replication and innate immune evasion and those interactions may also impact nsp5 function. Therefore, blocking these interactions could dampen viral replication. A recent study looked at interaction of 26 of 29 SARS-CoV-2 viral proteins with host proteins and showed that the catalytically dead nsp5 (C145A) interacts with proteins involved in the response to oxidative stress and mitochondrial matrix proteins [126]. Since nsp5 may cleave proteins that it interacts with and/or have short-lived interactions, these interactions may be difficult to assess by mass spectrometry. The proteins identified in the study are probably an underestimate of the host proteins that nsp5 is interacting with. Additional yeast two-hybrid studies have shown that nsp5 interacts with other nsps, including nsp7 and nsp8 in SARS-CoV [127] or nsp7, nsp9 and nsp12 in PRRSV [128]. Since these studies were done with mature proteins, these interactions should be independent of cleavage and represent interactions that can be blocked using small molecules.

Several groups have shown that CoV nsp5 proteases cleave host targets in innate immune pathways. For example, porcine deltacoronavirus (PDCoV), porcine epidemic diarrhoea virus (PEDV) and FIPV nsp5 proteases cleave the nuclear factor-κB (NF-κB) essential modulator (NEMO) to antagonize type I IFNs [129–131]. PDCoV also cleaves STAT2 to inhibit activation of IFN-stimulated genes [132]. Recent studies with SARS-CoV-2 showed that nsp5 cleaved IRF3 and two innate immune proteins, NLRP12 and TAB1 [133]. It is likely that nsp5 may target other host proteins for cleavage, which may provide a unique and novel avenue for development of nsp5 inhibitors. Fragment-based design is a technique that is now being used to identify allosteric inhibitors [134–137]. For the HIV protease (PR), this approach was used and identified two allosteric sites that bound to PR and prevented access to the active site [136]. It was not determined whether these locations could block binding to other proteins. However, the fragment-based design technique has been used to disrupt allosteric interactions between the protease and helicase domains of hepatitis C virus NS3 [138]. It would be interesting to see what interactions could be disrupted using this method on CoV nsp5 proteases.

## Conclusions

With the emergence of three coronaviruses in the last 20 years that cause significant disease and mortality, there remains a critical need for therapeutics for current and future emerging coronaviruses. As the SARS-CoV-2 pandemic approaches 25 million cases worldwide, the ability to have effective tools to limit COVID-19 disease and arrest the spread of this pandemic are paramount to a return to normality. Coronavirus protease nsp5 remains a key target for therapeutic design efforts and renewed interest should be given to find novel conserved structural and functional features of the protease that may be exploited. We have highlighted in this review an array of features that have and have not been extensively explored for therapeutic targets. It is our hope that an effective therapeutic with broad-spectrum activity against the nsp5 protease of a majority of coronaviruses can be developed to respond.
